# The *Chlamydia trachomatis* Extrusion Exit Mechanism Is Regulated by Host Abscission Proteins

**DOI:** 10.3390/microorganisms7050149

**Published:** 2019-05-25

**Authors:** Meghan Zuck, Kevin Hybiske

**Affiliations:** 1 Department of Medicine, Division of Allergy and Infectious Diseases, Center for Emerging and Reemerging Infectious Disease (CERID), University of Washington, Seattle, WA 98109, USA; mzuck@uw.edu; 2 Division of Infectious Diseases and Immunity, School of Public Health, University of California, Berkeley, CA 94720, USA

**Keywords:** *Chlamydia trachomatis*, extrusion, abscission

## Abstract

The cellular exit strategies of intracellular pathogens have a direct impact on microbial dissemination, transmission, and engagement of immune responses of the host. *Chlamydia* exit their host via a budding mechanism called extrusion, which offers protective benefits to *Chlamydia* as they navigate their extracellular environment. Many intracellular pathogens co-opt cellular abscission machinery to facilitate cell exit, which is utilized to perform scission of two newly formed daughter cells following mitosis. Similar to viral budding exit strategies, we hypothesize that an abscission-like mechanism is required to physically sever the chlamydial extrusion from the host cell, co-opting the membrane fission activities of the endosomal sorting complex required for transport (ESCRT) family of proteins that are necessary for cellular scission events, including abscission. To test this, *C. trachomatis* L2-infected HeLa cells were depleted of key abscission machinery proteins charged multivesicle body protein 4b (CHMP4B), ALIX, centrosome protein 55 (CEP55), or vacuolar protein sorting-associated protein 4A (VPS4A), using RNA interference (RNAi). Over 50% reduction in extrusion formation was achieved by depletion of CHMP4B, VPS4A, and ALIX, but no effect on extrusion was observed with CEP55 depletion. These results demonstrate a role for abscission machinery in *C. trachomatis* extrusion from the host cell, with ALIX, VPS4A and CHMP4B playing key functional roles in optimal extrusion release.

## 1. Introduction

*Chlamydia trachomatis* is an obligate intracellular bacterium that elicits a major public health burden worldwide [1,2]. *C. trachomatis* exits host cells by two mutually exclusive pathways that are dependent on distinct host-based mechanisms: extrusion and lysis [3]. Lysis is a destructive process, culminating in the release of free infectious bacteria. Extrusion is a packaged release of *Chlamydia*, that leaves the original host cell intact and often with a residual chlamydial inclusion. Extrusion is also highly conserved across chlamydiae, including divergent chlamydial species and *C. trachomatis* ocular, urogenital and lymphogranuloma venereum (LGV) serovars, which suggests important roles in chlamydial pathogenesis [4].

The pinching of the chlamydial inclusion just prior to extrusion is a result of actin polymerization on the cytosolic face of the inclusion, myosin-II-based contraction, Rho GTPase and Rho kinase (ROCK) activation, and the chlamydial inclusion membrane protein CT228 [3,5,6]. Specific inhibition of RhoA, the integral regulator of furrow ingression in cytokinesis, arrested extrusion at the final ‘pinching’ stage of exit [3]. However, the identity of the cellular machinery that function downstream of this stage, to sever the fully formed extrusion from the host cell membrane, remains to be defined. These data, coupled with the previously described disruptive effects of *C. trachomatis* infection on cell division, points to a mechanism whereby host cell division signaling pathways are hijacked by *Chlamydia* to facilitate escape from its intracellular niche [7,8,9,10].

Abscission is the final stage of cytokinesis, and consists of coordinated spatiotemporal regulation of dedicated proteins in order to ensure even chromosome segregation and cytoplasm distribution of daughter cells [11,12]. The critical proteins responsible for regulating the timing of abscission are the ESCRT proteins [13,14,15]. The ESCRT pathway mediates membrane scission in a number of cellular processes, and is targeted by several intracellular pathogens for facilitating their exit from host cells, for example the budding of enveloped viruses [16,17,18,19,20,21,22,23].

Current literature proposes that the ESCRT-III complex, of which subunit CHMP4B is crucial, is the core machinery responsible for deforming membranes through oligomerization of multiple subunits with shared structural homology [24]. As abscission of the two daughter cells proceeds, the intercellular bridge narrows, and the volume of microtubules within these region decreases by microtubule severing and depolymerization [25,26]. CHMP4B is recruited to the cell midbody by the accessory proteins ALIX and Tumor Suppressor Gene 101 (TSG101), which provide an ESCRT-III targeting platform at the midbody to facilitate membrane fission [12,27]. ALIX and TSG101 are in turn recruited to the midbody of the intercellular bridge by the interaction with CEP55 [28]. During the final stage of abscission, the ESCRT-III depolymerization factor VPS4 accumulates at the intercellular bridge by binding the ESCRT-III subunits [26]. Finally, the intercellular bridge is severed, enabling the release of the two daughter cells. Depletion of any of these core proteins leads to cytokinetic failure, as demonstrated by the increase in multinucleated cells across cell monolayers [28].

In this study, we tested the role of the important host abscission proteins ALIX, VPS4, CHMP4B, and CEP55 in facilitating extrusion formation. These proteins were selected based on their central roles in abscission, their parallel requirements for virus budding from host cells, and their characterized localizations at or near the midbody during mammalian cell abscission. Significant reduction in *Chlamydia* extrusion production were measured in cells depleted of CHMP4B, VPS4, and ALIX, but not CEP55. Furthermore, the localizations of ALIX and VPS4 were found to be distributed adjacent to chlamydial inclusions at late stages of infection, consistent with their functional involvements. Collectively, these results show that host abscission machinery are important components of the host-based mechanism that mediates *Chlamydia* extrusion from host cells. 

## 2. Materials and Methods

### 2.1. Cell Culture, Chlamydia Propagation and Infections

HeLa 229 and McCoy cells were grown in RPMI 1640 media supplemented with 10% FBS (HyClone, Thermo Fisher Scientific, Rockford, IL, USA) and 2 mM L-glutamine (HyClone), and cultured at 37 °C with CO_2_. For all microscopy experiments, cells were subcultured and plated onto chambered coverglass slides (Lab-Tek II; Nunc, Rochester, NY, USA), or glass bottom culture dishes (MatTek, Ashland, MA, USA) or 6-well and 24-well plates (BD Falcon). *C. trachomatis* serovar L2 (LGV 434/Bu) was propagated in L929 cells grown in suspension culture or HeLa cells grown in T75 flasks and purified as previously described [29]. Chlamydial elementary bodies (EBs) were isolated by sonic disruption of L929 suspensions and purification by centrifugation. The final bacterial pellet was resuspended in sucrose phosphate buffer (SPG; 5 mM glutamine, 0.2 M sucrose. 0.2 M phosphate buffer) and stored at −80 °C.

### 2.2. Extrusion Isolation

Green fluorescent protein (GFP)-expressing *C. trachomatis* serovar L2 was grown in semiconfluent HeLa cells for 72 h, or in McCoy cells for 48 h in RPMI supplemented with 10% FBS, L-glutamine and cycloheximide (2 µg/mL) [30]. Media on cell monolayers was removed, rinsed, and new media added to infected cultures at 72 hpi or 48 hpi (for McCoy cells). Infected cell cultures were allowed to proceed with infection in new media for 2–4 h to endogenously collect extrusions, then media was centrifuged at 75× g for 5 m, followed by removal of supernatant and a second centrifugation spin at 1200 rpm for 5 m. The extrusion pellet was immediately resuspended in fresh growth media. To enumerate the number of extrusions obtained from a cell monolayer, resuspended extrusions were stained with Hoechst (1:2000, Molecular Probes) for 5 min at 25 °C, plated as 20-µL drops onto glass slides and imaged immediately on a Nikon inverted fluorescence microscope. Intact extrusions were identified as having chlamydial inclusions, lacking nuclei and being of appropriate size [31].

### 2.3. Immunofluorescence and Live Fluorescence Microscopy

All live and immunofluorescence microscopy was performed on a Nikon Eclipse Ti inverted fluorescence microscope. Image capturing was obtained using a Hamamatsu camera controller C10600 and Volocity imaging software, version 6.3 (PerkinElmer; Waltham, MA, USA). Infected cells were fixed in 3.7% paraformaldehyde (Ted Pella) for 15 m, then permeabilized with 0.1% Triton-X-100 (Fisher), blocked with 1% BSA-PBS (Fisher), and stained. Antibodies/dyes were obtained from the following sources: mouse anti-ALIX antibody from Santa Cruz biotechnology (Dallas, TX, USA), rabbit anti-VPS4 from Sigma-Aldrich (St. Louis, MO, USA), mouse anti-CEP55 from Santa Cruz Biotechnology, rabbit anti-CHMP4B from Santa Cruz Biotechnology, CellLight ER-RFP from Thermo Fisher (Waltham, MA, USA), Phalloidin 633, donkey anti-goat 488 from Invitrogen (Waltham, MA), DAPI, goat anti-mouse 488 from Thermo Fisher, anti-GFP 488, FM4-64 from Molecular Probes (Eugene, OR, USA), mouse anti-*C. trachomatis* LPS donated by Bob Suchland (University of Washington, WA, USA). Staining of abscission proteins: HeLa cells were seeded onto glass chamber slides and fixed with paraformaldehyde 24 h after seeding to stain for antibody localization of CHMP4B, VPS4, CEP55 and ALIX proteins. All abscission proteins are stained with antibodies labeled by GFP and nuclei is displayed by DAPI staining.

### 2.4. Image Processing and Analysis

Three-dimensional image stacks were further processed in Volocity by performing illumination correction (in *z* dimension) and deconvolution (25 iterations). Individual *xy* and *xz* slices were obtained from image stacks for figure assembly. Three-dimensional opacity renderings of fluorescent image stacks were generated in Volocity. Minor retouching of all micrographs—for example, color assignment, contrast adjustment, RGB merges and cropping—were performed with Volocity and Photoshop CS6 (Adobe). Photoshop was used to assemble all figures into their final form.

### 2.5. RNA Interference

ON-TARGET plus pre-designed siRNA oligonucleotides were purchased from Dharmacon (Lafayette, CO, USA). HeLa cells grown in six-well plates or glass chamber slides were transfected twice with 20 nM siRNA duplexes at 24 h intervals using Lipofectamine RNAi MAX. Cells were harvested and analyzed after an additional 24 h except where specifically noted. HeLa cells were plated on day 0, infected early on Day 1, left to recover for several hours at 37 °C, then treated with siRNA at the end of Day 1. This initial knockdown was followed with a second knockdown on Day 2 (at 24 hpi). Subsequent extrusion isolation and other downstream assays were performed on Day 3 (at 48 hpi) (Table 1). 

### 2.6. Real Time Quantitative PCR

Uninfected HeLa cells, HeLa cells infected with *C. trachomatis* L2, and cells transfected with siRNA, were scraped from six-well dishes and pelleted by low speed centrifugation. RNA isolation was performed on ice by Qiagen RNEasy kit instructions. RNA concentrations were determined by spectrophotometry, and cDNA synthesis was performed using BioRad iscript cDNA synthesis kit. Samples were stored at 4 °C until needed for reverse transcription polymerase chain reaction (RT-PCR) analysis. RT-PCR was performed using SSOAdvanced SYBR green reagents. The relative abundance of each gene was calculated using the ΔΔCt method, and data were normalized to glyceraldehyde 3-phosphate dehydrogenase (GAPDH) expression. Primer sequences were obtained from published literature [32,33,34] (Table 2). 

### 2.7. Cell Multinucleation Assay

HeLa cells were plated onto glass chamber slides to reach 30–50% confluence at 24 hours (day of siRNA treatment). Knockdown was achieved by siRNA treatment on Days 1 and 2. On Day 2, approximately 4 h following siRNA treatment, live cells were stained with Hoechst to visualize host nuclei, and FM4-64 to visualize plasma membranes. Microscopy was performed at 60X magnification, with a minimum of 10 images per treatment group and a minimum of 100 cells per treatment. Assays were performed at least three independent times. For all images, cells with two or more nuclei were positively scored by manual counting. 

### 2.8. Statistical Analysis

Statistical evaluation of data was performed by calculating the standard error of the mean (SEM) or using linear regression, one way, or two-way analysis of variance (ANOVA). P-values <0.05 (*) were considered statistically significant. P-values of <0.001 (**), <0.0001 (***), and <0.00001 (****) were marked as indicated. Calculations were performed in Prism (GraphPad) and Microsoft Excel.

## 3. Results

### 3.1. Abscission Protein Distributions in Dividing HeLa Cells

Previous work demonstrated that the extrusion exit mechanism of *Chlamydia* harbored mechanistic, kinetic, and morphological similarities with the abscission stage of cellular cytokinesis [3]. The ESCRT accessory proteins ALIX and TSG101 have been shown to be recruited to the midbody of the cellular intercellular bridge through interactions with CEP55 [28]. The ESCRT-III subunit CHMP4B is in turn recruited by ALIX and TSG101, which collectively provide a platform at the midbody to facilitate membrane fission [12,24,27]. During the final stage of abscission, the ESCRT-III depolymerization factor VPS4 severs the intercellular bridge [26]. These proteins are localized and active at or near the midbody of the intercellular bridge during abscission, and we hypothesized they may play functional roles in the release of chlamydial extrusions.

**Figure 1:**
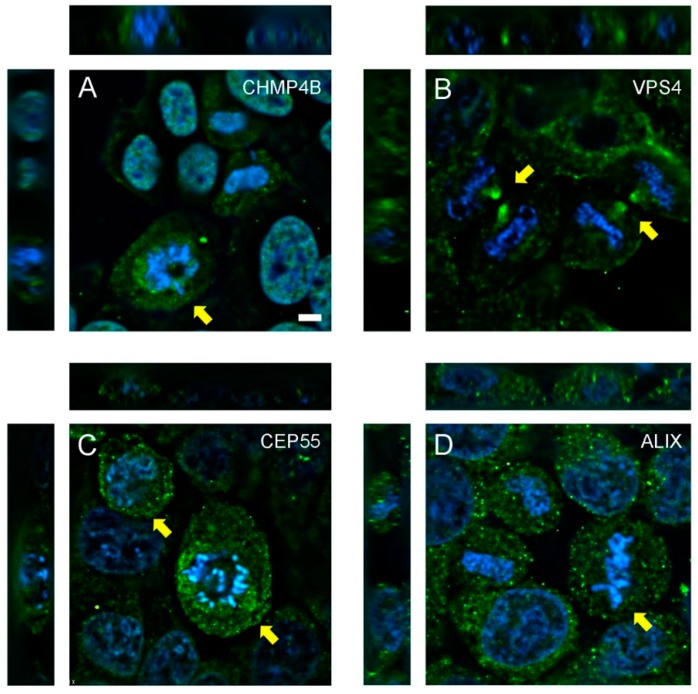
Localization and recruitment of abscission proteins in HeLa cells. HeLa cells were seeded onto glass chamber slides and fixed after 24 h, then stained with antibodies to (**A**) CHMP4B, (**B**) VPS4, (**C**) CEP55, or (**D**) ALIX. Proteins (green) and DAPI-labeled nuclei (blue) are shown. Z-stacking was performed to confirm localization and recruitment of proteins during various stages of mitosis, including abscission. Yellow arrows highlight specific recruitment or upregulation of abscission proteins within dividing cells. Scale bar = 10 µm.

CHMP4B was observed in the nucleus of resting HeLa cells, but displayed a transient, punctate, juxta-nuclear localization in dividing cells (Figure 1A, yellow arrows), similar to ESCRT recruitment dynamics of retroviral budding [35,36]. VPS4 localized to the edges of the recently severed intercellular bridge following abscission (Figure 1B, yellow arrows). CEP55 staining revealed no distinct spatial distribution, however, the amount of CEP55 was elevated slightly in dividing cells (Figure 1C, yellow arrows). ALIX elicited a punctate staining pattern in dividing cells (Figure 1D). This distribution did not differ from cells in interphase that were not undergoing mitosis, and ALIX was not recruited to a specific subcellular location during any stage of cell division.

### 3.2. Depletion of Abscission Proteins in HeLa Cells

**Figure 2:**
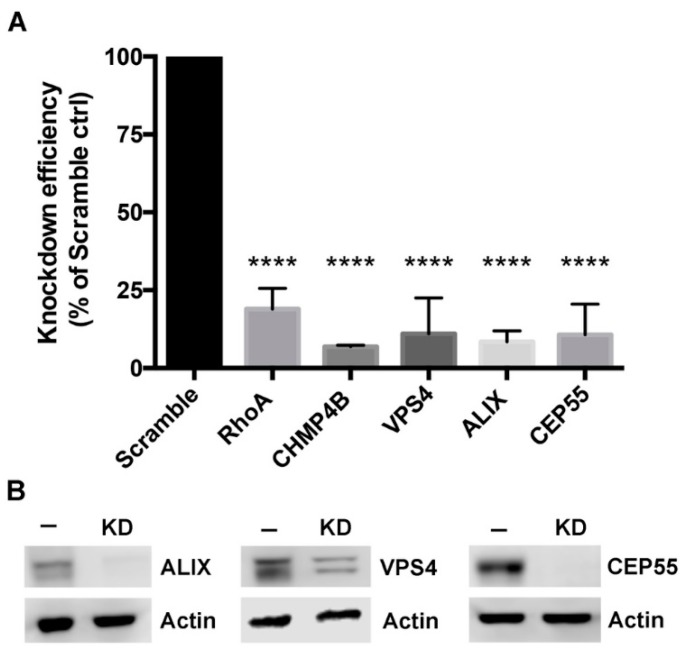
Knockdown of host abscission proteins. (**A**) Knockdown efficiency was measured by RT-PCR; values for each target are presented as a percent of gene expression compared to cells treated with a non-targeting siRNA (Scramble) control. Data points show mean ± SEM, n = 3. Statistics were performed using one-way ANOVA with Dunnett’s multiple comparisons post-test. **** denotes a p value < 0.0001. (**B**) HeLa cells treated with scramble siRNA (−), or treated with siRNA (KD) for VPS4, ALIX, or CEP55, and actin control.

Knockdown of candidate abscission proteins in HeLa cells was accomplished using RNA interference (RNAi). Cells were treated with either control (scrambled) siRNA, or siRNA oligonucleotides that specifically targeted RhoA, CHMP4B, VPS4, ALIX, or CEP55 (at t = 0 h and t = 24 h), and knockdown efficiency was analyzed at 48 h by RT-PCR. Strong knockdown of each host target was measured, with depletion efficiencies calculated to be 81% ± 7% for RhoA, 93% ± 0.5% for CHMP4B, 89% ± 11% for VPS4, 91% ± 3% for ALIX, and 89% ± 10% for CEP55 (Figure 2A). Western blots were performed to confirm protein depletion of ALIX, VPS4, and CEP55, with complete knockdown achieved for ALIX and CEP55 and a partial depletion of VPS4 (Figure 2B). The VPS4 western blot displayed two bands, which are likely to be the two isoforms VPS4a and VPS4b (Figure 2B) [13]. 

### 3.3. Multinucleated Phenotypes Observed Following siRNA Transfection

**Figure 3:**
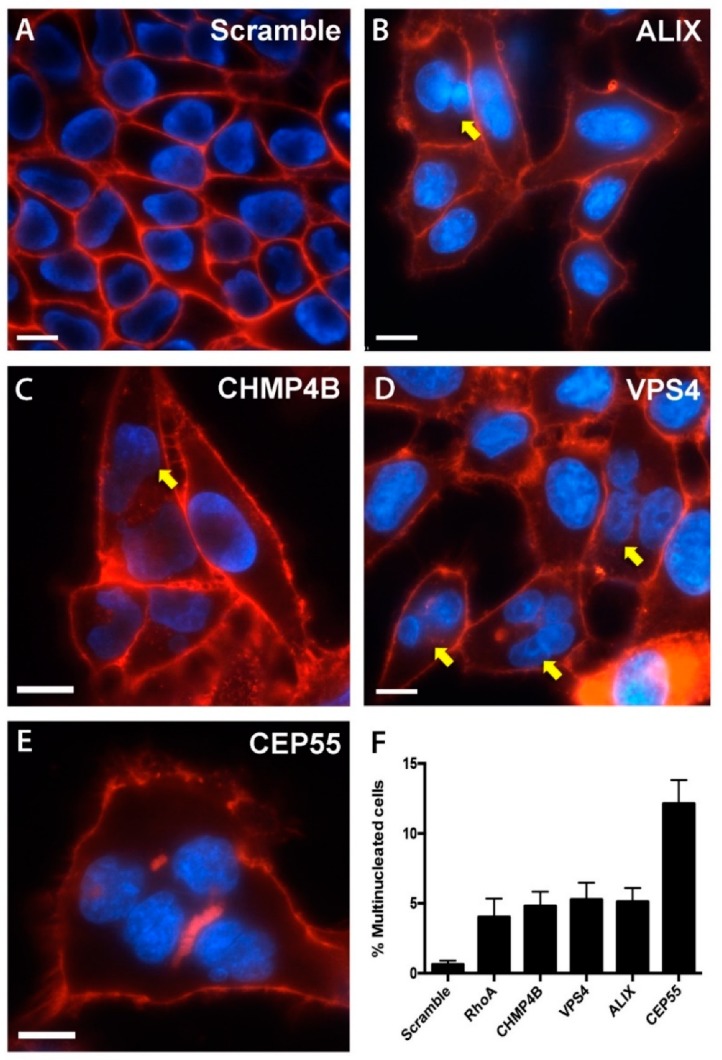
Knockdown of abscission proteins results in multinucleated cells. Representative images of HeLa cells treated with: (**A**) Scramble siRNA, (**B**) ALIX siRNA, (**C**) CHMP4B siRNA. (**D**) VPS4 siRNA, (**E**) CEP55. Following siRNA treatments, cells were fixed, stained, and imaged by fluorescence microscopy at 60X magnification. Yellow arrows depict cells with multiple nuclei. (**F**) Quantitative analysis of cell multinucleation of a minimum of 100 cells per image, across 30 images per treatment, with data displayed as the percentage of cells with two or more nuclei. Plasma membranes were labeled with FM4-64 (red), nuclei were labeled with DAPI (blue). A minimum of 10 image fields were taken for each treatment, n = 3, scale bar = 10 μm.

Failure of cells to effectively complete abscission can result in multiple or fragmented nuclei, or single and multiple midbodies within dividing cells. To examine these unique abscission defects, cells were depleted of a single abscission protein, and imaged by live fluorescence microscopy. Cells treated with a non-targeting scramble siRNA had a low percentage of multinucleated cells, 0.62% of cells in the monolayer (Figure 3A,F). Depletion of ALIX, CHMP4B, VSP4, and CEP55, all resulted in an increase in the number of multinucleated cells: 5.1%, 4.8%, 5.3%, and 12.1%, respectively (Figure 3B–F). An increase in cell multinucleation was also found in cells depleted of the small GTPase RhoA, which functions upstream of abscission protein recruitment during cytokinesis (Figure 3F). Even with robust protein depletion, this relatively low percent of multinucleated cells has been previously reported, and is likely due to strong mitotic defects masking downstream abscission defects [13]. Additional abscission failure phenotypes were also commonly observed in our analysis, including intact cell midbodies and fragmented nuclei, confirming that abscission failure occurred following knockdown of individual abscission targets.

### 3.4. Chlamydial Inclusion Morphology and Infectivity Not Affected by Depletion of Abscission Proteins

**Figure 4:**
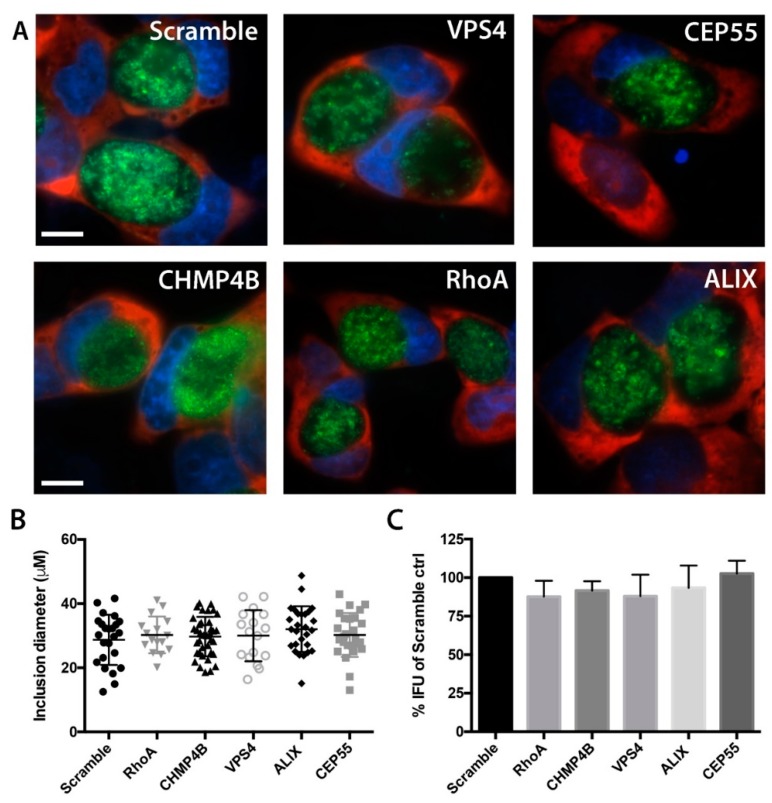
Primary *C. trachomatis* infections are not affected by abscission protein RNAi. (**A**) Morphologies of *C. trachomatis* inclusions at 48 hpi following siRNA treatments with a nontargeting control (Scramble), VPS4, CEP55, CHMP4B, RhoA, and ALIX. Images were acquired at 60X magnification. GFP-expressing *C. trachomatis* (green), mCherry-HeLa cells (red), and DAPI-labeled nuclei (blue) are labeled. Scale bar = 10 μm. (**B**) Inclusion diameters of all inclusions in 10 images per host target knockdown. Diameter measurements were performed from nucleus side of the inclusion outward. (**C**) IFU analysis of *Chlamydia*-infected HeLa cells following knockdown with abscission targets. For graphs in B and C, n = 3, and no significance was measured using one way ANOVA with Dunnett’s multiple comparisons test in inclusion diameter or IFU compared to Scramble control.

To determine whether the depletion of abscission proteins by RNAi had any adverse effects on early stages of *Chlamydia* infection, we measured the diameters of primary *C. trachomatis* inclusions in cells treated with siRNA compared to cells treated with a nontargeting siRNA control. mCherry-HeLa cells [37] were treated with siRNA, infected with GFP-expressing *C. trachomatis* L2, and inclusion diameters were measured by live cell fluorescence microscopy. Inclusions were determined by the areas containing GFP-expressing bacteria, and diameters of inclusions were calculated at 48 hpi on a per cell basis by imaging software analysis. No decreases in inclusion growth were detected for any of the siRNA treatments (Figure 4A,B), indicating that chlamydial growth proceeded normally in cells lacking expression of these proteins. The productive generation of infectious EB was also determined by inclusion forming unit (IFU) assays. Compared to scramble control, there were no statistically significant changes in complete chlamydial growth for any siRNA treatment (Figure 4C). Together, these results provide confidence that measurements of late stage events in *Chlamydia*-infected cells targeted with these siRNA treatments are not confounded by upstream effects of these protein depletions on *Chlamydia* developmental growth. 

### 3.5. Depletion of Abscission Proteins Partially Inhibited Extrusion Production

**Figure 5:**
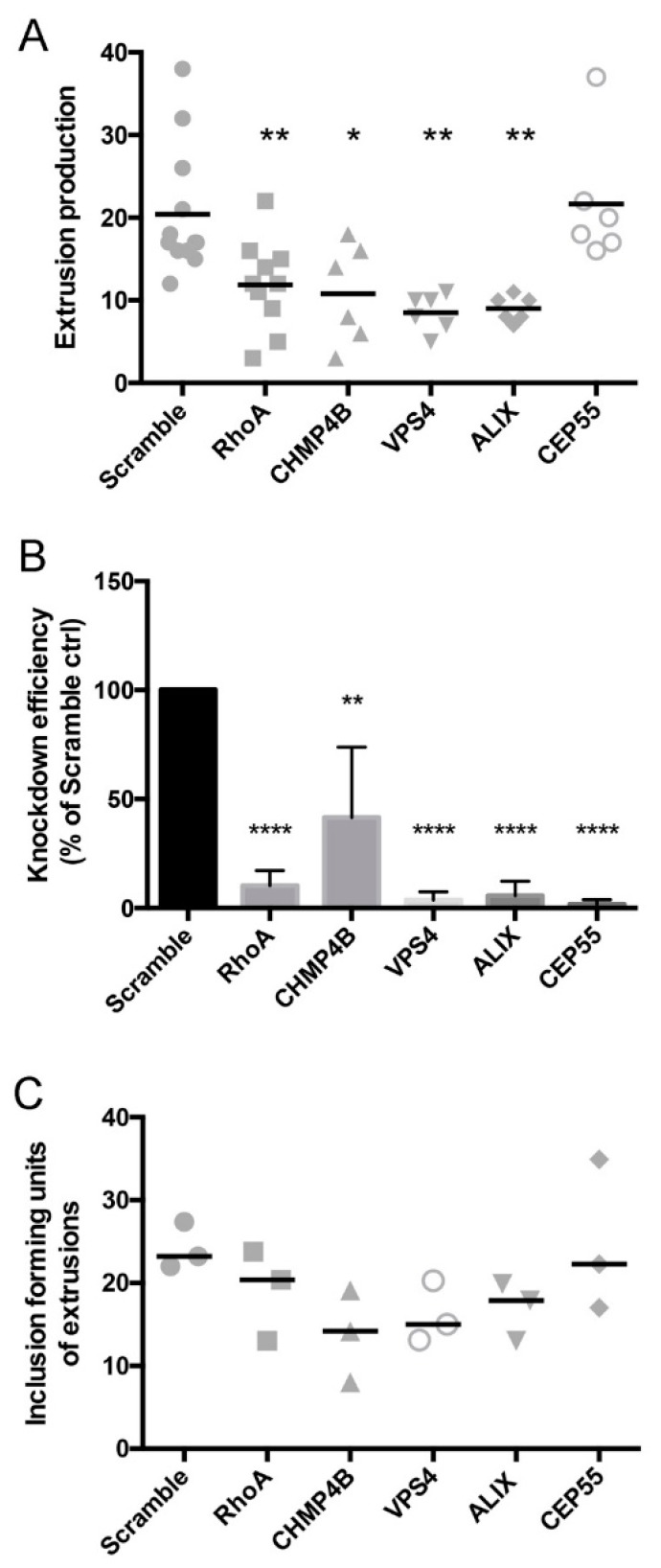
*Chlamydia* extrusion release is dependent on host abscission proteins. (**A**) The release of *C. trachomatis* containing extrusions from siRNA-treated HeLa cells was determined measured by microscopic analysis of centrifugation-enriched extrusion suspensions. (**B**) Knockdown efficiencies of siRNA treatments on *Chlamydia*-infected HeLa cells, as measured by quantitative RT-PCR. (**C**) IFU determinations on isolated extrusion suspensions were performed by plating sonicated extrusion samples onto fresh HeLa cells, followed by IFU analysis at 48 hpi. Data points show mean ± SEM, n = 3. Statistics were performed using One-Way ANOVA with Dunnett’s multiple comparisons post-test. **** denotes p value < 0.0001, ** denotes p value < 0.01, * denotes p value < 0.05.

We next examined the impact of abscission protein depletion on the release of *Chlamydia* extrusions from HeLa cells. Extrusions were harvested from *Chlamydia*-infected, siRNA-treated cells at 48 hpi, and quantified by live fluorescence microscopy using published procedures [31]. Depletion of individual abscission targets resulted in significant reductions in detached or released extrusions: 42% ± 8% for RhoA knockdown cells, 47% ± 15% for CHMP4B knockdown cells, 58% ± 9% for VPS4A knockdown cells, and 56 ± 7% for ALIX knockdown cells (Figure 5A). No effect of CEP55 depletion was measured on extrusion release, as it was indistinguishable from control cells (Figure 5A). In addition to these effects on extrusion production as measured by live fluorescence microscopy, we additionally performed IFU analysis on extrusion suspensions to verify that reduced numbers of bacteria were released by cells upon siRNA treatments. For all siRNA treatments except CEP55, depletion of host abscission proteins resulted in significantly less IFU, confirming that fewer extrusions were produced by cells with disrupted abscission machinery (Figure 5B).

### 3.6. Distributions of Abscission Proteins on Late-Stage Chlamydial Inclusions

**Figure 6:**
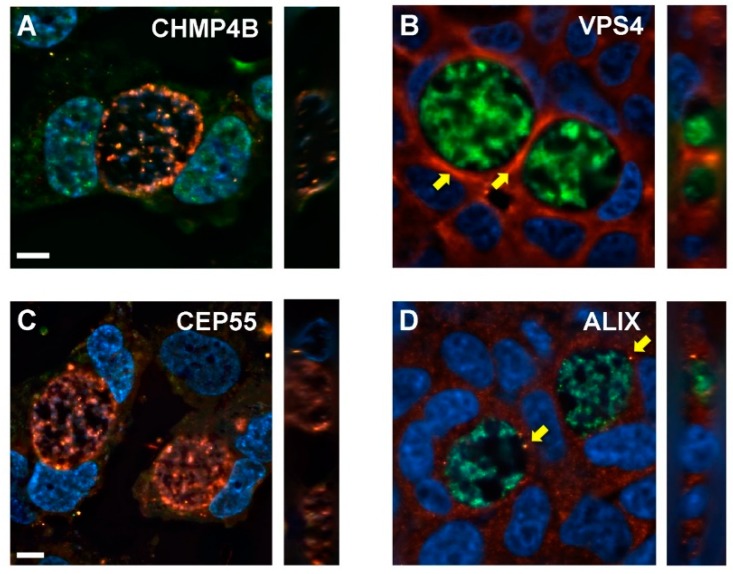
Localizations of abscission proteins in late-stage *Chlamydia*-infected HeLa cells. HeLa cells were infected with GFP- or mCherry-expressing *C. trachomatis* L2, transfected with siRNA against CHMP4B, VPS4, CEP55, or ALIX, and fixed at 48 hpi for analysis of abscission protein distributions in relation to late-stage inclusions. (**A**) anti-CHMP4B (green), mCherry-*C. trachomatis* L2 (red), and DAPI-stained nuclei (blue). (**B**) anti-VPS4 (red), GFP-*C. trachomatis* L2 (green), and DAPI-stained nuclei (blue). Yellow arrows denote regions of increased VPS4 staining around the inclusion. (**C**) anti-CEP55 (green), mCherry-*C. trachomatis* L2 (red), DAPI-stained nuclei (blue). (**D**) anti-ALIX (red), GFP-*C. trachomatis* L2 (green), DAPI-stained nuclei (blue). Yellow arrows indicate punctate localization of ALIX adjacent to chlamydial inclusion. Z-stacking was performed to confirm localization to inclusion inside infected cells. Scale bars = 10 μm.

During cytokinesis, the proteins VPS4, CHMP4B, ALIX, and CEP55 localize to the midbody to allow for abscission of the two tethered daughter cells. We hypothesized that similar to this redistribution for cytokinesis, these critical abscission proteins may also be recruited to chlamydial inclusion membranes prior to and/or during extrusion. To explore this, *Chlamydia*-infected HeLa cells were analyzed by immunofluorescence microscopy for the recruitment or upregulation of abscission proteins during late-stage infection (46 hpi). Analysis of CHMP4B and CEP55 showed no distinct localizations of these proteins with respect to chlamydial inclusions (Figure 6A,C). The distribution of VPS4 appeared to be most intense at regions adjacent to inclusions (Figure 6B), however we cannot exclude the possibility that reduced cytoplasmic space contributes to this patterning. ALIX staining within infected cells routinely resulted in discrete puncta that were closely associated with chlamydial inclusions (Figure 6D), consistent with functional data for ALIX in downstream extrusion detachment from host cells. 

## 4. Discussion

Collectively, the results presented within this work demonstrate an important functional role for host abscission machinery in the endogenous detachment, or release, of chlamydial extrusions from host cells. All previous mechanistic information for the *Chlamydia* extrusion pathway has been focused on early aspects of the mechanism; thus, the findings of the present study fill an important knowledge gap in our understanding of *Chlamydia* cell to cell spread. The depletion of ALIX, CHMP4B, and VPS4A—proteins with characterized functions in cellular abscission—resulted in significant reductions of naturally-released extrusions, and concurrent reductions in IFU from extrusion suspensions collected from these treated cells. The extrusions produced by siRNA-treated cells showed no phenotypic differences in extrusion appearance or size. Although strong knockdowns were achieved for our targets, the depletions of individual abscission targets were incapable of completely inhibiting the release of extrusions. These results contrast with requirements for abscission proteins in mammalian cells in the absence of infection, where each ESCRT-III protein plays a significant functional role [13], and may therefore suggest that abscission machinery is involved, but not essential for extrusion production. Alternatively, there may be redundancy in the pathway that mediates the detachment of extrusions from host cells; future investigations should attempt to disrupt multiple abscission proteins and regulatory factors. 

CEP55 was found to be dispensable for extrusion release. As CEP55 regulates the localization of ALIX and TSG101, both of which are critical to successful severing of daughter cells during cytokinesis, this is somewhat surprising. It is possible that there is redundancy in recruitment of proteins to ensure efficient escape of *Chlamydia*, or that duplicate or multiple knockdown of abscission proteins is necessary to completely abrogate the extrusion mechanism altogether. 

The involvement of these key abscission proteins parallels similar requirements for viral exit strategies, including HIV budding from the host cell [18,38], and therefore abscission proteins may be a common target of intracellular pathogens faced with the challenge of exiting cells by a membrane-mediated route. These findings are even more intriguing in light of recent data, demonstrating an interaction between ESCRT-I protein TSG101, and a chlamydial effector protein, CT619 [39]. TSG101, with its interaction partner ALIX, are crucial to normal kinetics and progression of cell abscission. Without any known function of TSG101 in chlamydial development in vitro, it is possible that these proteins are targeted by *Chlamydia* specifically to facilitate exit via extrusion [39].

## Figures and Tables

**Table 1:** siRNA oligonucleotide sequences.

Protein	Target Sequence
Human CEP55 (55165)	CUGAGUGAAUUUCGAAGAA
Non-targeting pool	UGGUUUACAUGUCGACUAA
	UGGUUUACAUGUUGUGUGA
	UGGUUUACAUGUUUUCCUA
Human ALIX	CAGAUCUGCUUGACAUUUA
Human VPS4	CCACAAACAUCCCAUGGGU
Human CHMP4B	CCAUCGAGUUCCAGCGGGA
	AGAAGAGUUUGACGAGGAU
	CGGAAGAGAUGUUAAGCAA
	UGGAAAGGGUCGACUGGUU

**Table 2:** Primer sequences used.

Gene Name	Sequence 5’ to 3’
GAPDH fwd	GGTGCTGAGTATGTCGTGGA
GAPDH rev	CGGAGATGATGACCCTTTTG
ALIX fwd	GACGCTCCTGAGATATTATGATCAG
ALIX rev	ACACACAGCTCTTTTCATATCCTAAGC
VPS4 fwd	GGAAGACGGAAGGCTACTCG
VPS4 rev	AGGGGCCACAGACCTTTTTG
CEP55 fwd	GGAGGGCAGACCATTTCAGAG
CEP55 rev	AGGCTTCGATCCCCACTTAC
RhoA fwd	GTGGATGGAAAGCAGGTAGAG
RhoA rev	TAACATCGGTATCTGGGTAGGA
CHMP4B fwd	GGAGAAGAGTTTGACGAGGATG
CHMP4B rev	CTGTTTCGGGTCCACTGATT
